# Microorganism-Driven 2,4-D Biodegradation: Current Status and Emerging Opportunities

**DOI:** 10.3390/molecules29163869

**Published:** 2024-08-15

**Authors:** Shao-Fang Chen, Wen-Juan Chen, Haoran Song, Mingqiu Liu, Sandhya Mishra, Mohamed A. Ghorab, Shaohua Chen, Changqing Chang

**Affiliations:** 1 State Key Laboratory for Conservation and Utilization of Subtropical Agro-Bioresources, Guangdong Province Key Laboratory of Microbial Signals and Disease Control, Engineering Research Center of Biological Control, Ministry of Education, South China Agricultural University, Guangzhou 510642, China; 2 College of Plant Protection, South China Agricultural University, Guangzhou 510642, China; 3 Department of Environmental Microbiology, Babasaheb Bhimrao Ambedkar University, Lucknow 226025, India; 4 The Office of Chemical Safety and Pollution Prevention, U.S. Environmental Protection Agency (EPA), Washington, DC 20460, USA

**Keywords:** 2,4-D, herbicide, biodegradation, metabolites, degradation pathways, molecular mechanisms

## Abstract

The herbicide 2,4-dichlorophenoxyacetic acid (2,4-D) has been widely used around the world in both agricultural and non-agricultural fields due to its high activity. However, the heavy use of 2,4-D has resulted in serious environmental contamination, posing a significant risk to non-target organisms, including human beings. This has raised substantial concerns regarding its impact. In addition to agricultural use, accidental spills of 2,4-D can pose serious threats to human health and the ecosystem, emphasizing the importance of prompt pollution remediation. A variety of technologies have been developed to remove 2,4-D residues from the environment, such as incineration, adsorption, ozonation, photodegradation, the photo-Fenton process, and microbial degradation. Compared with traditional physical and chemical remediation methods, microorganisms are the most effective way to remediate 2,4-D pollution because of their rich species, wide distribution, and diverse metabolic pathways. Numerous studies demonstrate that the degradation of 2,4-D in the environment is primarily driven by enzymatic processes carried out by soil microorganisms. To date, a number of bacterial and fungal strains associated with 2,4-D biodegradation have been isolated, such as *Sphingomonas*, *Pseudomonas*, *Cupriavidus*, *Achromobacter*, *Ochrobactrum*, *Mortierella*, and *Umbelopsis*. Moreover, several key enzymes and genes responsible for 2,4-D biodegradation are also being identified. However, further in-depth research based on multi-omics is needed to elaborate their role in the evolution of novel catabolic pathways and the microbial degradation of 2,4-D. Here, this review provides a comprehensive analysis of recent progress on elucidating the degradation mechanisms of the herbicide 2,4-D, including the microbial strains responsible for its degradation, the enzymes participating in its degradation, and the associated genetic components. Furthermore, it explores the complex biochemical pathways and molecular mechanisms involved in the biodegradation of 2,4-D. In addition, molecular docking techniques are employed to identify crucial amino acids within an alpha-ketoglutarate-dependent 2,4-D dioxygenase that interacts with 2,4-D, thereby offering valuable insights that can inform the development of effective strategies for the biological remediation of this herbicide.

## 1. Introduction

**Figure 1:**
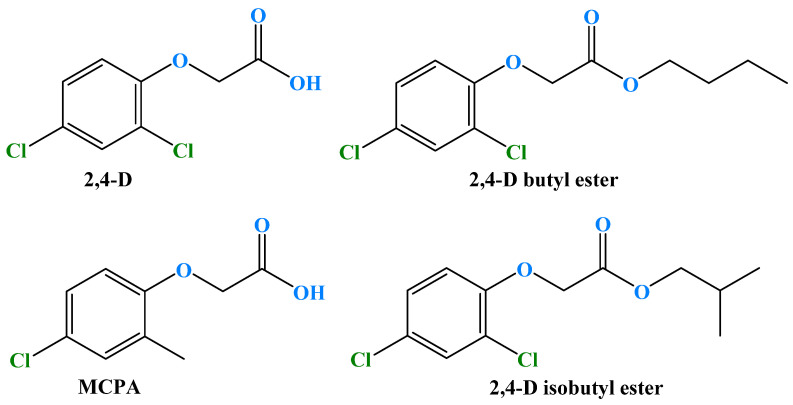
The chemical structures of 2,4-D and its derivants. 2,4-D: 2,4-dichlorophenoxyacetic acid; 2,4-D butyl ester: butyl 2,4-dichlorophenoxyacetate; 2,4-D isobutyl ester: isobutyl 2,4-dichlorophenoxyacetate; MCPA: 2-methyl-4-chlorophenoxyacetic acid. They all belong to the phenoxyalkanoic acid herbicide group.

2,4-dichlorophenoxyacetic acid (2,4-D) has a well-established history of use, known for its effectiveness in managing weeds, wide range of weed control capabilities, and cost-effectiveness [1]. 2,4-D belongs to the phenoxyalkanoic acid (PAA) herbicide group. Varieties within this herbicide family are classified based on the substituents present on the benzene ring, the positions of substitution, and the groups attached to OCH-COOH. Examples of these varieties include butyl 2,4-dichlorophenoxyacetate (2,4-D butyl ester), isobutyl 2,4-dichlorophenoxyacetate (2,4-D isobutyl ester), and 2-methyl-4-chlorophenoxyacetic acid (MCPA), represented by distinct chemical formulas, as depicted in Figure 1. The utilization of 2,4-D is widespread globally, with significant usage observed in markets such as the United States, South America, China, Europe, and Russia. In the United States specifically, annual consumption of 2,4-D amounts to around 46 million pounds, with agricultural activities accounting for 66 percent of this usage, ranches for 23 percent, and residential homeowners for 11 percent [2]. During the Vietnam War, the United States military utilized a herbicidal defoliant known as “Agent Orange”, which consisted of a mixture of 2,4-D and 2,4,5-trichlorophenoxyacetic acid (2,4,5-T), in order to eliminate the thick jungle vegetation that served as camouflage for Vietnamese soldiers [3,4,5]. In China, annual application of 5000 to 8000 tons of 2,4-D butyl ester is utilized for the purpose of controlling weeds in crop cultivation [6]. Annually in Argentina, approximately 2200 metric tons of 2,4-D herbicide are applied for the growth of various crops [7,8,9,10,11]. It is crucial to note that the widespread and consistent application of 2,4-D has the potential to foster weed resistance, leading to environmental contamination and potential health risks to humans. 

**Figure 2:**
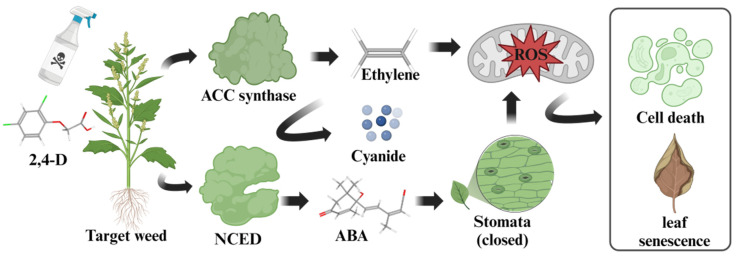
The proposed mechanism and pattern of action of the auxin herbicide 2,4-D at concentrations exceeding the optimal levels within weeds [21,22,23]. Upon 2,4-D’s activation of the ethylene synthesis process via the 1-aminocyclopropane-1-carboxylic acid (ACC, a precursor to ethylene biosynthesis) pathway, cyanide builds up as a side product. Under such circumstances, elevated levels of ethylene can impede plant growth, and cyanide can be detrimental to the plants’ health. Within the bud tissue, the gene for 9-*cis*-epoxycarotenoid dioxygenase (NCED), a key enzyme that mediates the regulation of abscisic acid (ABA) biosynthesis in plants, is overexpressed, triggering the generation of ABA. This hormone triggers the closure of the stomata, thereby limiting the plant’s transpiration process. Concurrently, there is an excessive production of reactive oxygen species (ROS). Furthermore, ABA, together with ethylene, promotes leaf senescence, impairs chloroplast function, and undermines the structural integrity of the vascular system. As a consequence, this may result in tissue dehydration and decomposition, culminating in the demise of the plant.

2,4-D is a man-made auxin herbicide that functions as a weed control agent by interfering with the hormone-mediated signaling pathways in weeds [12,13,14]. Figure 2 depicts the mechanism and pattern of action of the auxin herbicide 2,4-D in weeds. The herbicide 2,4-D functions similarly to the natural plant hormone indole-3-acetic acid (IAA). When applied at suitable concentrations, it triggers the activation of genes linked to auxins, specifically those essential for the production of ethylene and abscisic acid (ABA). These compounds lead to the suppression of cell division, hastening leaf senescence, and consequently inhibiting plant growth, ultimately resulting in the death of the targeted weed [15,16]. Regarded as a fundamental component within the category of herbicides, this systemic herbicide is extensively employed for the management of both annual and perennial broadleaf weeds in agricultural and non-agricultural contexts, such as parks, roads, grasslands, turf areas, and golf courses [17,18,19,20]. 2,4-D is a significant component in modern weed control practices across agricultural and non-agricultural domains, underscoring the necessity for increased attention towards understanding the environmental persistence of 2,4-D residues.

Because of its high solubility in water and low capacity for soil adsorption, 2,4-D is commonly found in surface water and can potentially migrate to groundwater via the hydrological cycle [24,25]. Concentrations of 2,4-D spanning from a few to several hundred micrograms per liter are regularly identified in surface water, groundwater, and soil [26,27,28]. Research findings indicate that 2,4-D has been detected in the water bodies of Argentina, prompting an evaluation of surface water quality using physicochemical and ecotoxicological parameters [29,30]. In a study conducted in 2007 to assess the presence of herbicides in urban rivers and streams throughout Canada, the herbicide 2,4-D was frequently detected at sampling locations, exhibiting an average concentration of 172 ng/L [31]. Urban wastewater in Australia has also been found to contain 2,4-D at a level of 3 μg/L [32]. 2,4-D demonstrates a significant tendency to leach, as evidenced by the detection of increased concentrations in groundwater samples taken from specific shallow soil layers [33,34,35]. While a small fraction of 2,4-D is taken up by the targeted weeds, the majority of it permeates the soil and water systems [36]. Given its persistence across various environmental conditions, 2,4-D contributes to environmental contamination [2]. Residues of 2,4-D present significant health risks to non-target organisms as a result of their application [37,38,39,40]. Additionally, the structure of the soil microbial community typically experiences significant alterations following the application of 2,4-D [41,42,43]. 2,4-D is listed as a Class 2B toxic herbicide by the World Health Organization [44]. 

**Figure 3:**
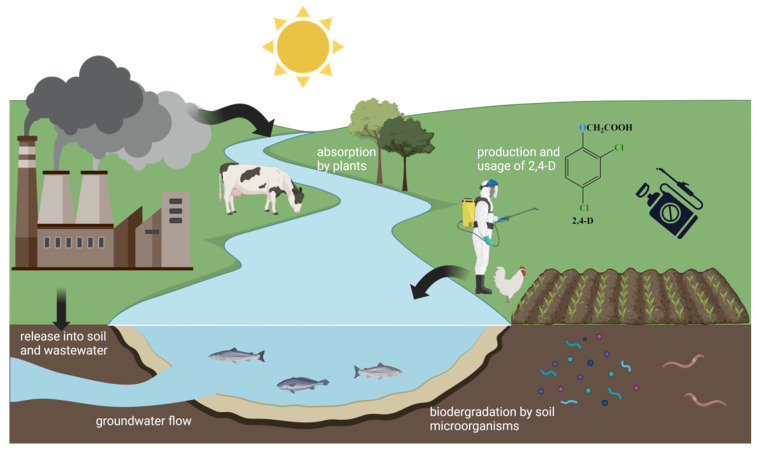
The occurrence of 2,4-D in the environment and its fate. A very small fraction of 2,4-D reaches the target weeds, while the remainder is released into the soil and water environments. As it flows with runoff and groundwater, it poses a threat to non-target flora and fauna, and more seriously, it create notable hazards for human health. Decomposition and metabolism by soil microorganisms is the primary method for the transformation of residual 2,4-D in the natural environment.

Various techniques, such as photocatalysis, ion exchange, redox reactions, and electrochemical processes, are available for the removal of the herbicide 2,4-D and its detrimental byproduct, 2,4-dichlorophenol (2,4-DCP), from the environment [45,46,47]. However, these methods are often expensive and require specialized equipment, materials, and chemicals, rendering them unfeasible for broad application [48]. The utilization of microorganisms for the degradation or reduction of xenobiotic pollutants, a process known as bioremediation, is widely regarded as an environmentally sustainable and ecologically advantageous method [49]. This process typically begins with the adsorption of herbicide molecules onto microbial cell surfaces, followed by their transport across the cell membrane and subsequent metabolism. Microbes produce enzymes that catalyze the breakdown of herbicide molecules through various biochemical pathways, such as oxidation, reduction, or hydrolysis [50,51,52]. Additionally, the application of genomics and proteomics has facilitated the discovery of novel genes and proteins that contribute to the biodegradation process. Metagenomic analysis of microbial communities in contaminated environments has also unveiled the diversity of the catabolic pathways present, which can be harnessed for enhanced bioremediation strategies. Synthetic biology approaches, such as the design of microbial consortia with complementary metabolic functions, are also promising for addressing the complexity of herbicide mixtures in the environment. Moreover, the progress in creating highly effective engineered bacteria capable of degrading pollutants using synthetic biology and genetic engineering techniques has significantly enhanced the process of bioremediation for organic contaminants [53]. In summary, the mechanisms and molecular advancement of herbicide bioremediation have advanced significantly, offering insights into the biodegradation process and paving the way for the development of effective and targeted biotechnological solutions to mitigate herbicide pollution. Figure 3 illustrates the occurrence of 2,4-D in the environment and its fate. 

To date, there has been much research on the biodegradation of 2,4-D and its degradation genes; however, there is currently no general overview of the biodegradation pathways and molecular mechanisms of 2,4-D. Here, this review provides a comprehensive analysis of the recent progress in elucidating the degradation mechanisms of 2,4-D, including the microbial strains responsible for its degradation, the enzymes participating in its degradation, and the associated genetic components. Furthermore, this review discusses the existing problems in the current research on 2,4-D bioremediation, thereby providing new ideas for the removal of 2,4-D pollution from the environment.

## 2. Toxicity

2,4-dichlorophenoxyacetic acid (2,4-D) is frequently employed for the control of broadleaf weeds in agricultural environments, particularly in the production of crops such as wheat, rice, corn, sorghum, and sugarcane [54,55]. Nonetheless, more than 90% of the herbicide 2,4-D that is administered ultimately infiltrates the soil and water systems, with a minimal portion effectively reaching the intended weed targets [56,57]. 2,4-D exhibits varying degrees of persistence in diverse natural settings, characterized by a degradation half-life spanning from 20 to 312 days [58]. Due to its high water solubility and low adsorption coefficient, the herbicide 2,4-D is commonly detected in both surface and groundwater sources, posing notable environmental and health risks [59,60]. Non-target organisms may be exposed to 2,4-D through various pathways, which could result in toxicological effects that are dependent on factors such as the amount of exposure, frequency of exposure, and the organism’s susceptibility [61]. 

The World Health Organization (WHO) recommends a maximum allowable concentration of 2,4-D in drinking water of 20 micrograms per liter [62]. 2,4-D is recognized for its cytotoxic properties and is classified as a potential genotoxic agent. It is categorized as a moderately hazardous Class II herbicide by the World Health Organization (WHO), and the International Agency for Research on Cancer (IARC) has classified it as potentially carcinogenic to humans, assigning it to Group 2B [63,64]. In addition, extensive research has confirmed its potential to cause neurotoxic, hepatotoxic, immunosuppressive, and teratogenic effects [65,66]. Soloneski and colleagues conducted a study to examine the genotoxic impacts of 2,4-D through an assessment of the mitotic indices, sister chromatid exchange (SCE), and cell cycle advancement in human plasma leukocyte cultures (PLCs) and whole blood cultures (WBCs). The results from the WBCs indicated a significant increase in SCE frequency following exposure to 2,4-D concentrations ranging from 10 to 50 µg/mL. Conversely, no significant effect on SCE frequency was noted in the PLCs within the same concentration range, indicating a differing susceptibility of WBCs and PLCs to the genotoxic effects of 2,4-D. Treatment with 25 and 50 µg/mL of 2,4-D resulted in a notable deceleration in cell proliferation in the WBCs, while the application of 100 µg/mL in the PLCs disrupted cell cycle progression [67]. Ruiz et al. utilized the Single-Cell Gel Electrophoresis (SCGE) method for analysis and assessed the long-term genotoxicity of 2,4-D in *Cnesterodon decemmaculatus* fish, demonstrating that exposure to 2,4-D over an extended period induces primary DNA damage [68,69,70]. The liver serves as a crucial organ responsible for processing, detoxifying, and eliminating harmful substances from the body, making it a key target of environmental pollutants. In a study conducted by Curi et al., alterations in the liver tissue of *Physalaemus albonotatus* tadpoles were observed following exposure to 2,4-D [71]. These changes included the formation of vacuoles in their hepatocytes, sinusoidal enlargement, blood vessel dilation, and an increase in melanin-containing macrophages. This research indicates that prolonged exposure to 2,4-D could endanger the survival of these tadpoles, induce various morphological abnormalities, and lead to hepatic dysfunction [71]. Another study examined the hepatotoxic impacts of 2,4-D on rats by assessing serum enzyme levels as markers of liver injury. Rats were orally administered the 2,4-D herbicide “Désormone lourd” daily for a period of 4 weeks. The results of the investigation revealed that 2,4-D triggers hepatotoxic effects in rats [72]. Reactive oxygen species (ROS) can inhibit an organism’s antioxidant capacity, leading to oxidative stress, which is associated with chronic diseases over the long term [73,74]. A study has shown that exposure to 2,4-D corresponds with a moderate increase in urinary levels of 8-OHdG (a biomarker of DNA oxidation damage) and 8-isoPGF (a product of lipoprotein peroxidation). The researchers posit that oxidative stress triggered by 2,4-D could be instrumental in the development of cancer and a range of chronic illnesses [75]. Moreover, the harmful effects of 2,4-D on male fertility have been validated by research findings which indicate that exposure to this herbicide raises the risk of infertility [76,77,78]. In brief, the presence of 2,4-D residues poses significant risks to non-target organisms and the ecological system, underscoring the importance of researchers being deeply concerned about the removal of 2,4-D from the environment.

## 3. 2,4-D-Degrading Microorganisms

The isolation and identification of effective 2,4-D degradation strains play a crucial role in the bioremediation of residual environmental contamination with 2,4-D. Microorganisms present in the soil are primarily responsible for the degradation of 2,4-D in the environment [79]. Numerous microbial strains exhibiting the degradation of 2,4-D, displaying favorable degradation properties and exhibiting resilience across a broad pH spectrum, have been documented. Various strains and associated degradation scenarios have been compiled and presented in Table 1. Dai et al. observed that in soil microcosms into which *Novosphingobium* sp. strain DY4 was introduced, the herbicide 2,4-D at a concentration of 200 mg/L was nearly entirely eliminated within a span of 5–7 days, resulting in a degradation efficiency of 96% [80]. Under specific conditions including an inoculation volume of 3% (*v*/*v*), a pH of 7.0, and a temperature of 30 °C, *Cupriavidus pinatubonensis* BJ71 demonstrated the capability to degrade 99% of 2,4-D within a 6-day cultivation period, starting with an initial concentration of 350 mg/L. Additionally, strain BJ71 exhibited the capacity to degrade fluroxypyr and quizalofop, representing the first documented instance of a 2,4-D-degrading bacterium harboring the *tfdA* gene, enabling the metabolism of these two herbicides [81]. 

To enable microorganisms capable of degrading specific herbicides to survive in the natural environment and compete with other species, several factors must be considered. First and foremost, the impact of environmental conditions on the survival and activity of these microbes is crucial. Therefore, alkali-tolerant degrading strains are isolated from contaminated sites, possessing the ability to adapt to the environmental stress of high pH values. Secondly, it is essential to understand how these degrading strains interact with other organisms in the environment, competing for survival resources. Additionally, the success of bioremediation projects requires rigorous monitoring and assessment of the remediation process. Isolating bacteria that can survive and remain active in extreme environments is more valuable for the bioremediation and remediation of contaminated sites [110,111,112]. Specifically, while many degrading bacteria have poor survival capabilities or low activity in some alkaline environments, such as alkaline mining tailings, saline–alkali lands, industrial accident sites, and the rubble of abandoned factory buildings, the domesticated strain *Cupriavidus oxalaticus* X32 can still perform well in degrading at a pH of 10.5 [82]. Moreover, compared to two alkaline-tolerant 2,4-D degrading bacteria, *Corynebacterium humireducens* MFC-5 and *Delftia acidovorans* P4a, which require additional carbon sources, X32 can grow using 2,4-D as its sole carbon and energy source [86,87,88]. Currently, the predominant group of bacteria identified in the environment for their ability to degrade 2,4-D are classified within the Gamma and Beta subdivisions of the proteobacteria phylum [113]. In addition, some degrading bacteria are members of the class Alpha proteobacteria, including the genera *Bradyrhizobium* and *Sphingomonas* [114,115]. There is considerable evidence indicating that the specific influence of 2,4-D’s selective pressure leads to notable changes in the composition of soil microbial communities [116,117,118]. In the presence of native soil microorganisms, the likelihood of introduced non-native strains persisting and operating is minimal. Therefore, employing naturally existing 2,4-D-degrading microbes presents a feasible approach to remediating polluted sites. Furthermore, research has demonstrated that cellular and genetic biological enhancements can significantly improve the on-site degradation of organic pollutants in contaminated soils [119,120,121,122]. 

## 4. Bacterial and Fungal Degradation Pathways of 2,4-D

Scholars have categorized 2,4-D-degrading bacteria into three classes [123]. The first class comprises certain symbiotic, rapidly growing bacteria from Beta proteobacteria, which possess the gene *tfdA* for the alpha-ketoglutarate-dependent dioxygenase that catalyzes the initial step in the degradation of 2,4-D [124]. The members of the second class belong to the *Pseudomonas* genus within Gamma proteobacteria [86]. The third class of 2,4-D-degrading bacteria are predominantly sourced from pristine soil environments that have not been contaminated, such as volcanic soils [125,126]. Characterized by a slower growth rate, they encompass the Alpha proteobacteria group of *Bradyrhizobia* and *Sphingomonas* and possess a *tfdA*-like or *cadA* gene, which can express enzymes related to the degradation of the herbicide PAA [87,127]. 

**Figure 4:**
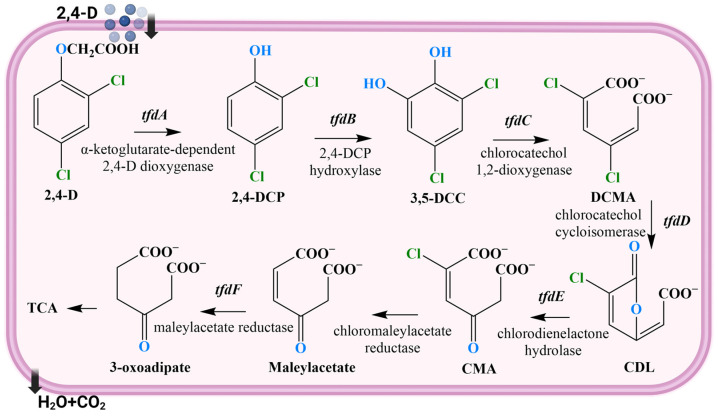
The 2,4-D degradation pathways proposed in *Cupriavidus necator* JMP134 [88,113,129].

Studies have shown that employing microorganisms for the degradation of remaining 2,4-D in the ecosystem is regarded as an environmentally sustainable, efficient, and economical approach [124,125,126,127]. Bacterial enzymes work in a stepwise manner to break down harmful halogenated aromatic compounds into typical cellular metabolites [128]. Detailed 2,4-D biodegradation pathways have been proposed, as shown in Figure 4. In *Cupriavidus necator* JMP134, the *tfdA* gene encodes an α-ketoglutarate-dependent 2,4-D dioxygenase that catalyzes the cleavage of the ether bond in 2,4-D, converting it into 2,4-DCP. Subsequently, the 2,4-DCP hydroxylase encoded by *tfdB* hydroxylates the phenolic compound to form 3,5-dichlorocatechol (3,5-DCC), which undergoes ortho or meta cleavage under the action of the chlorocatechol 1,2-dioxygenase encoded by *tfdC*, resulting in the formation of 2,4-dichloro-*cis*,*cis*-muconate (DCMA). The intermediate is further metabolized into *cis*-2-dichlorodiene lactone (CDL) by the chlorocatechol cycloisomerase encoded by *tfdD*. CDL is transformed into 2-chloromaleylacetate (CMA) through the catalytic activity of the chlorodienelactone hydrolase encoded by *tfdE*, which is then reduced into maleylacetate by chloromaleylacetate reductase [113]. Finally, 3-oxoadepate is generated and enters the tricarboxylic acid (TCA) cycle via the maleylacetate reductase encoded by *tfdF* [88,129]. 

**Figure 5:**
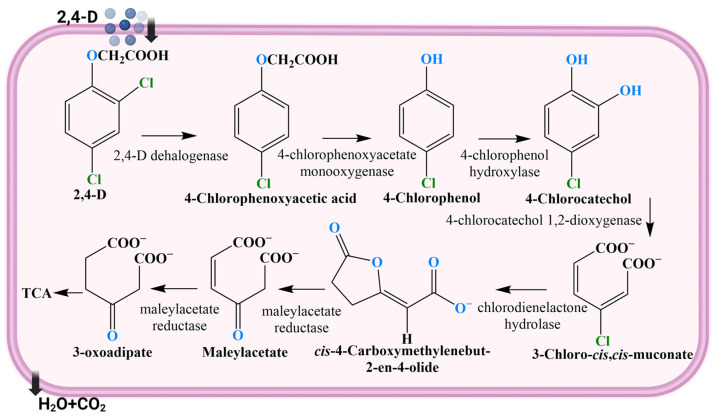
The 2,4-D degradation pathways proposed in *Azotobacter chroococcum* [131,132,133].

In an alternative metabolic pathway for 2,4-D shown in Figure 5, the reductive dehalogenation at the 2-position chlorine occurs before the removal of the acetoxyl group [130,131]. It typically involves the reductive dechlorination of 2,4-D by a dehalogenase enzyme, leading to the generation of 4-chlorophenoxyacetic acid, which is then transformed into 4-chlorophenol by the action of 4-chlorophenoxyacetate monooxygenase. Following this, 4-chlorophenol is hydroxylated by 4-chlorophenol hydroxylase into 4-chlorocatechol, which is subsequently converted into 3-chloro-*cis*,*cis*-muconate and *cis*-4-carboxymethylenebut-2-en-4-olide as a result of the enzymatic reactions involving 4-chlorocatechol 1,2-dioxygenase and chlorodienelactone hydrolase. In the end, maleylacetate reductase catalyzes the stepwise conversion of this intermediate first into maleylacetate and subsequently into 3-oxoadipate, which then becomes part of the TCA cycle [132]. It is important to recognize that certain isolated bacterial colonies may lack the full suite of enzymes necessary for the total mineralization of 2,4-D, potentially only possessing those that target the primary compound. As a result, a consortium of microorganisms can achieve a more efficient and comprehensive breakdown of the herbicide [133]. 

**Figure 6:**
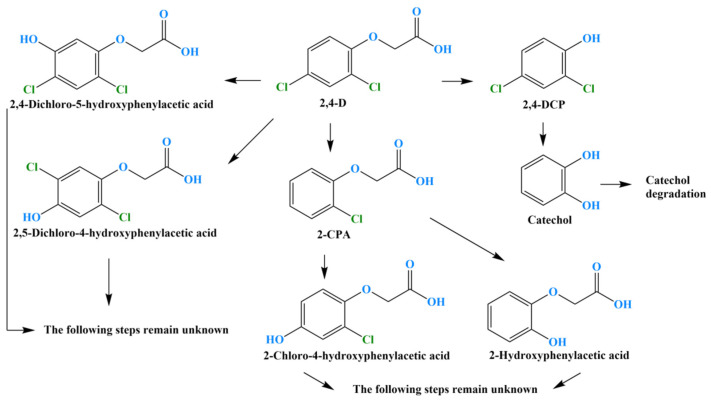
The 2,4-D degradation pathways proposed in *Aspergillus niger* [120,121,122].

While fungi from the divisions Ascomycota, Mucoromycota, and Basidiomycota are known for their potent ability to break down organic pollutants, there is scant information available regarding the enzymatic mechanisms that facilitate this biotransformation process [102,134]. In tests involving 90 fungal strains for the degradation of 2,4-D and 2,4-DCP, *Mortierella isabellina* and *Aspergillus penicilloides* showed superior performance, whereas *Mucor generensis* and *Chrysosporium pannorum* were particularly adept at breaking down 2,4-DCP [103]. The fungal degradation pathways of 2,4-D typically involve the transformation of numerous complex metabolic products [104,105,135,136]. In the degradation of 2,4-D by *Aspergillus niger*, dechlorination occurs first to produce 2-chlorophenoxyacetic acid (2-CPA), followed by hydroxylation reactions to form 2-chloro-4-hydroxyphenylacetic acid and 2-hydroxyphenylacetic acid, as shown in Figure 6. In another strain of *Aspergillus niger*, the metabolism of 2,4-D primarily involves the hydroxylation of the aromatic ring, resulting in the production of 2,5-dichloro-4-hydroxyphenylacetic acid and 2,4-dichloro-5-hydroxyphenylacetic acid [137,138,139]. Shailubhai et al. have suggested that there is a 2,4-DCP pathway in the degradation of 2,4-D by *Aspergillus niger*, with catechol being among the subsequent products formed [140]. 

The determination of intermediary metabolic byproducts resulting from the microbial breakdown of 2,4-D, along with the elucidation of the degradation pathways of 2,4-D in microorganisms, serves to uncover the molecular-level interactions between degrading enzymes and the herbicide [141]. This information not only enhances our understanding of the degradation process but also establishes a theoretical framework for the advancement of effective biodegradation strategies for residual herbicides. 

## 5. Genes and Enzymes Associated with the Degradation of 2,4-D

**Figure 7:**
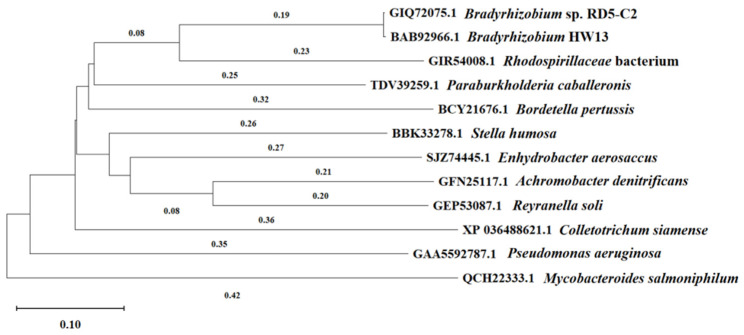
A neighbor-joining phylogenetic tree constructed based on the amino acid sequences of alpha-ketoglutarate-dependent 2,4-D dioxygenases from *Bradyrhizobium* sp. strain HW13 and other microbial strains (available from the NCBI nonredundant protein sequence database and the UniProtKB/Swiss-Prot database). The phylogenetic neighbor-joining tree was constructed using MEGA (version 11) software with 1000 bootstraps. Consensus sequences of every protein were aligned with ClustalW and trimmed to the same sizes. The number below the branch represents the branch length, which is calculated from the p-distance to show the evolutionary distance between different samples. The bar represents a 0.10 amino acid difference per site. The code preceding each species name is the NCBI accession number for the 2,4-D dioxygenase.

The classic 2,4-D degradation gene cluster *tfd* (*tfdABCDEF*) was initially identified in the plasmid pJP4 (AY365053) of *Cupriavidus necator* JMP134 (formerly known as *Wautersia eutropha*, *Ralstonia eutropha*, and *Alcaligenes eutrophus*) [89,142,143]. The *tfd* gene cluster metabolizes 2,4-D through the ortho cleavage pathway of chlorinated catechols. The genes *tfdABCDEF* are involved in the stepwise catabolic metabolism of 2,4-D, as mentioned above, while *tfdK* has the function of 2,4-D transport, and *tfdT* and *tfdR* encode regulatory factors necessary for the process [144,145]. The *tfdA* gene family consists of three classes of highly homologous sequences. The *tfdA* gene of *C. necator* JMP134 is identified as Class I, Class II is composed of *tfdA* sequences from the *Burkholderia* genus belonging to Beta proteobacteria, and Class III includes *tfdA* sequences from various genera of Beta and Gamma proteobacteria [123]. In *Sphingomonas* sp. strain TFD44, two chlorocatechol gene clusters were cloned by Thiel and colleagues. The first gene cluster, *tfdDRFCE*, includes all the genes required for converting 3,5-dichlorocatechol into 3-oxoadipate, as well as the regulatory gene *tfdR* from the LysR family. The second gene cluster *tfdC_2_E_2_F_2_* is incomplete, lacking a chlorocatechol cycloisomerase gene *tfdD*. Interestingly, their research results indicate that *tfdD* is involved in the degradation of 2,4-D, but the TFD44 strain has an additional chlorocatechol cycloisomerase gene, which allows the second gene cluster to still be expressed when growing with 2,4-D [90]. Remarkably, many studies have reported the diversity of *tfdA* gene sequences, and it has been proven that sequence diversity is not correlated with substrate specificity within the *tfdA*-like gene family [146,147]. The transposable elements IS1071 and ISThsp19 flanking the *tfd* gene cluster may be responsible for its transfer among bacteria and its widespread distribution in the environment [79,148]. Figure 7 is a neighbor-joining phylogenetic tree constructed based on the amino acid sequences of alpha-ketoglutarate-dependent 2,4-D dioxygenases from *Bradyrhizobium* sp. strain HW13 (BAB92966.1) and other species, and the results show that 2,4-D dioxygenases from the same genus of *Bradyrhizobium* exhibit the highest degree of similarity. Subsequently, the 2,4-D dioxygenase from *Bradyrhizobium* HW13 and that from the *Rhodospirillaceae* bacterium (GIR54008.1), both belonging to the Alpha proteobacteria class, show a high degree of similarity, indicating that the 2,4-D dioxygenases have a relatively close evolutionary relationship. 

**Figure 8:**
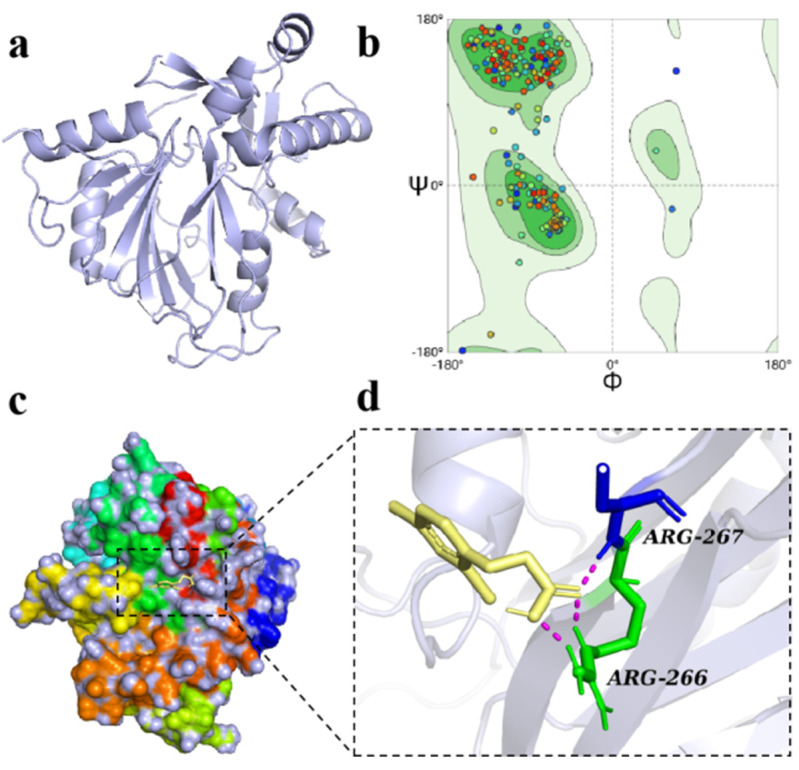
Prediction of the protein structure and a molecular docking model of an alpha-ketoglutarate-dependent 2,4-D dioxygenase (GenBank Accession Number 499491759) from Burkholderiaceae bacteria. (**a**) Cartoon structure of the 2,4-D dioxygenase; (**b**) Ramachandran plot of the 2,4-D dioxygenase; (**c**) Surface structure of the 2,4-D dioxygenase; (**d**) Molecular docking result. Arg 266 and Arg 267 as key amino acids in the 2,4-D dioxygenase.

Certainly, with the exception of gene cluster *tfd*, there are other genes that also possess the capacity to degrade 2,4-D. The 2,4-D degradation gene cluster, *cadRABKC*, discovered in *Bradyrhizobium* sp. strain HW13, is also capable of effectively metabolizing 2,4-D [91]. The *cadK* gene is believed to encode a 2,4-D transporter because it exhibits a high degree of sequence similarity to the 2,4-D transport protein gene *tfdK* from strain JMP134 [149]. Kitagawa et al. hypothesized that the *cadA*, *cadB*, and *cadC* genes encode the large, small, and ferredoxin subunits of 2,4-D oxygenase, respectively. Notably, unlike *tfdA*, the dioxygenase gene *cadA* is a member of the aromatic-ring-hydroxylating dioxygenase genes [150]. Hayashi et al. identified the *cadABCK* gene cluster in *Bradyrhizobium elkanii* USDA94, a model strain of soybean rhizobium, and demonstrated that the strain can degrade 2,4-D when *cadABCK* is forcibly expressed. *Escherichia coli* engineered to carry the *cadABCK* gene also showed degradation ability. Their research results indicate that the lack of degradative ability in the wild strain is due to the poor induction capability of 2,4-D and 2,4,5-T for degradative genes [92]. These alternative pathways can involve different initial steps or intermediate compounds compared to the *tfd*-mediated pathway. Aroxyalkanoate dioxygenases (ADDs) derived from *Sphingobium herbicidovorans* can efficiently degrade 2,4-D into nonherbicidal dichlorophenol and glyoxylate, which can confer field resistance to 2,4-D when expressed in soybeans and corn [151,152]. Using the Swiss-Model website, a three-dimensional structure model of the alpha-ketoglutarate-dependent 2,4-D dioxygenase from a strain of *Burkholderiaceae* bacteria was constructed, as shown in Figure 8a. The model’s Ramachandran-favored value reached 95.44%, with only 0.7% of the amino acid residues located in the disallowed region in white, well below the 10% safety threshold. Furthermore, the global model quality estimation (GMQE) value of this model is 0.94, exceeding the critical threshold of 0.5, indicating that this three-dimensional structure model is suitable for molecular docking analysis (Figure 8b). The 3D model file of 2,4-D was obtained from the PubChem website, and subsequent molecular docking was performed using the AutoDockTools (version 1.5.7). Figure 8c displays the spatial binding state of 2,4-D with the 2,4-D dioxygenase. When 2,4-D enters the binding pocket, the molecular docking results indicate that Arg266 and Arg267 in this 2,4-D dioxygenase form hydrogen bonds with the oxygen atoms on the carboxyl group of 2,4-D (Figure 8d). 

The outstanding fungal enzymes that have been explored as effective biodegradants are peroxidases, cytochrome P450 monooxygenases, and laccases, which catalyze the transformation of polluting compounds in enzymatic reactions [153,154,155,156,157]. Nguyen and colleagues have indicated that laccases play a significant role in the degradation of 2,4,5-T and 2,4-D by the fungus *Rigidoporus* sp. FMD21. Furthermore, their research has revealed that strain FMD21 partially relies on cytochromes P450 (CYPs) for involvement in the transformation pathways of 2,4-D and 2,4,5-T. However, it has not yet been determined which enzymes are responsible for the degradation of 2,4-D and 2,4,5-T in Ascomycota *Fusarium* sp. T1-BH.1 and *Verticillium* sp. T1-BH.2 [106]. In biodegradation, CYPs play a prominent role in the hydroxylation of aromatic compounds and the detoxification of these pollutants, such as the cleavage of aromatic rings, hydroxylation by elimination of the chlorine substituents, and hydroxylation of chloride substituents for migration [158]. These hydroxylation steps are crucial in the breakdown pathway for 2,4-D [159]. It is demonstrated that in the filamentous fungus *Umbelopsis isabellina* DSM 1414, cytochrome P450 plays a primary role in the metabolism of 2,4-D [160].

The presence of numerous genes and gene clusters responsible for degradation in microorganisms highlights the operational capacity of microbial communities, which can be harnessed in bioremediation approaches aimed at remedying herbicide-contaminated ecosystems. 

## 6. Conclusions and Future Perspectives

Anticipated future increases in the utilization of 2,4-D are expected due to the growing global demand for food resulting from population expansion. While 2,4-D currently serves a crucial function in both agricultural and non-agricultural weed management, it presents notable risks to environmental integrity, human well-being, and ecosystems. Microorganisms exhibit considerable potential for their involvement in biotechnological practices related to environmental remediation. However, employing them to biodegrade herbicides presents a variety of challenges. These encompass the tolerance of microorganisms to herbicidal toxins, the complexity of the metabolic pathways, the shifting dynamics within microbial communities, the need for supportive policies and regulations, the translation of technology from the lab to the field, and the influence of environmental factors like temperature, moisture, pH levels, and soil types. Therefore, a more intensive focus is needed on exploring a greater variety of tolerant degrading strains and identifying degradative gene resources. There is a need for heightened focus on the breakdown of 2,4-D by soil microorganisms, particularly in the underexplored realm of fungal resources. The metabolic pathways and enzymatic processes implicated in the fungal degradation of 2,4-D remain incompletely understood, necessitating further investigation in this domain. The isolation and examination of highly effective degrading strains, along with a comprehensive comprehension of the degrading enzymes and their genetic, pathway-related, and mechanistic aspects, are imperative for the development of efficient strategies aimed at detoxifying polluted sites. Additionally, policies and regulations must be established to encourage and support the research and application of bioremediation technology. 

The development of genetically engineered microorganisms capable of degrading the herbicide 2,4-D presents novel approaches to addressing environmental remediation of 2,4-D contamination and the ecological management of industrial wastewater. Progress in genetic engineering and the identification of genes conferring herbicide resistance have led several countries to approve genetically modified organisms resistant to these chemicals, particularly in the enhancement of herbicide tolerance in new varieties of cotton, soy, canola, corn, and other crops. The identification and characterization of degradation genes play a crucial role in expanding genetic diversity and are essential for reducing herbicide residues and introducing herbicide-resistant traits into genetically modified plants. 

## Figures and Tables

**Table 1:** Categories and sources of 2,4-D-degrading microorganisms and their degradation characteristics.

	Microorganisms	Sources	Degradation Efficiency	Initial 2,4-D Concentration	Incubation Conditions	Comments	References
	*Cupriavidus gilardii* T-1	A soybean field	100% in 20 h	100 mg/L	pH 7.0–9.0, 37–42 °C	Fast degradation of 2,4-D and its analogues highlights the potential for the use of *C. gilardii* T-1 in bioremediation of PAA herbicides.	[79]
	*Novosphingobium* DY4	A paddy-planted field site	More than 95% in 5–7 d	200 mg/kg	pH 7.2, 30 °C	DY4 contained the *TfdAα* gene (converting 2,4-D into 2,4-DCP).	[80]
	*Cupriavidus campinensis* BJ71	Soil	99.57% in 6 d	350 mg/L	pH 7.0, 30 °C	Strain BJ71 could also degrade quizalofop and fluroxypyr.	[81]
	*Cupriavidus oxalaticus* X32	Sewage sludge	100% within 3 d	500 mg/L	30 °C, pH 6.5	X32 still functioned at a pH of 10.5.	[82]
	*Delftia acidovorans* P4a	Concrete samples of herbicide plant	ND	ND	ND	A chromosomally located catabolic transposon carries genes for a complete 2,4-D degradation pathway.	[83]
	*Corynebacterium humireducens* MFC-5	MFC	ND	ND	pH 10.0	The reductions were greatly enhanced by the addition of quinones/humics serving as redox mediators.	[84]
	*Achromobacter* sp. QXH	A mixture of soil and activated sludge	ND	ND	ND	Bioaugmentation promoted the steady transformation of glucose-grown granules into 2,4-D-degrading sludge granules and fast establishment of 2,4-D degradation ability.	[85]
	*Burkholderia cepacia* DS-1, *Pseudomonas* sp. DS-2, *Sphingomonas paucimobilis* DS-3	Soil	69%, 73%, and 54% in 10 d, respectively	50 mg/L	30 ± 1 °C	Strains DS-1 and DS-2 may additionally possess the potential to metabolize 2,4-DCP.	[86]
	*Sphingomonas agrestis* 58-1	Soil	ND	ND	30 °C	Induced the overexpression of the *cadAB* genes using *E. coli* as the host strain.	[87]
	*Achromobacter xylosoxidans* subsp. *denitrificans* strain EST4002	Soil	ND	ND	ND	EST4002 contains plasmid pEST4011.	[88]
	*Variovorax paradoxus* TV1	Soil	ND	ND	ND	The PTV1 partial *tfdA* sequence showed 77% similarity to the archetypal *tfdA* gene sequence from *C. necator* JMP134.	[89]
	*Sphingomonas* sp. TFD44	Herbicide wastewater treatment facility	ND	ND	ND	Strain TFD 44’s *tfd_I_* gene cluster and pJP4’s *tfd_II_* gene cluster share some common characteristics.	[90]
	*Bradyrhizobium* sp. strain HW13	Hawaiian soil	ND	ND	30 °C	A new family of 2,4-D degradation genes, *cadRABKC*, was cloned and characterized.	[91]
	*Bradyrhizobium elkanii* USDA94	Soil	ND	ND	ND	The *cad* cluster in the ordinary root-nodulating *B. elkanii* USDA94 had the ability to degrade 2,4-D and 2,4,5-T.	[92]
	*Pseudomonas* sp. NJ 10, *Pseudomonas aeruginosa* NJ 15	Agricultural soil	96.6% and 99.8% in 20 d, respectively	100 mg/L	pH 7.0, 37 °C	The isolated strains possess phosphate-solubilizing capabilities.	[93]
	*Aeronomas hydrophila* IBRB-36 4CPA	ND	ND	ND	ND	Plasmid pAH36 contains a gene for chlorine-substituted phenoxyacetic acid catabolism.	[94]
	*Halomonas* sp. EF43	Alkaline lake sediment	ND	ND	30 °C, pH 10.0	Strain EF43 can stably maintain the pJP4 plasmid carrying the 2,4-D degradation genes.	[95]
	*Corynebacterium* sp. SOGU16, *Achromobacter* sp. SOGU11	Oil-contaminated soils	ND	ND	pH 7.2, 27 ± 2 °C	The optimum pH for dioxygenase-specific activities was between 7.6 and 8.0, and the temperature was between 30 and 35 °C.	[96]
	*Cupriavidus* sp. CY-1	Forest soil	100% within 72 h	500 mg/L	28 °C	Strain was isolated from uncontaminated soil.	[97]
	*Achromobacter* sp. LZ35	Soil	90% in 12 days	50 mg/L	30 °C, pH 8.0	The first report of an *Achromobacter* sp. strain that was capable of mineralizing both 2,4-D and MCPA.	[98]
	*Delftia* sp.	A polluted river	99.0% in 28 h	200 mg/L	28 °C	The ability of the strain to degrade and detoxify 2,4-D in synthetic wastewater across different aerobic reactors was assessed.	[99]
	*Rhodococcus ruber*, *Ochrobactrum anthropic*,	WWTP	In about 2 mo	100 mg/L	pH 7.0, 20 °C	The experiments identified *Rhodococcus* as the main genus biodegrading 2,4-D.	[100]
	*Cupriavidus pampae* CPDB6^T^	An agricultural soil	22% after 25 d	350 mg/L	30 °C	The strain was able to deamidate acetamide, which differentiated it from all other species of *Cupriavidus.*	[101]
Fungi	*Penicillium* sp.	Soil	29.80%	100 mg/L	30 °C	ND	[102]
	*Aspergillus penicilloides*, *Umbelopsis isabelina* (Former *Mortierella isabellina*)	Soil and decayed wood and walnut	52% and 46% after 5 d	100 mg/L	pH 4.5	Strain responses varied with the taxonomic groups and the chemicals tested.	[103]
	*Penicillium chrysogenum* CLONA2	Saltmine	ND	ND	pH 7.0	*Penicillium chrysogenum* in solid medium was able to grow at concentrations of up to 1000 mg/L of 2,4-D with sucrose.	[104]
	*Mortierella* sp.	Soil	32% within 1 h	250 μM	25 °C	ND	[105]
	*Rigidoporus* sp. FMD21, *Fusarium* sp. T1-BH.1, *Verticillium* sp. T1-BH.2	Decayed wood Soil	ND	ND	pH 6.0, 30 °C	Laccase is important for the degradation of 2,4-D; CYPs are involved in the pathway of 2,4-D transformation by *Rigidoporus* sp. FMD21.	[106]
	*Penicillium* sp.	Soil	ND	ND	28 °C	The first report of a *Penicillium* strain possessing the capability to degrade 2,4-D.	[107]
	*Umbelopsis isabellina* DSM1414	Soil	98% after 5 d	25 mg/L	28 °C	Enzymes responsible for 2,4-D degradation by this fungus include CYPs.	[108]
	*Trametes versicolor* (L.:Fr.) Pilát Mo008	*Crescentia alata*	100% in 850 h	1000 mg/L	pH 5.0, 25 °C	The strain presented activity of an enzymatic complex which was composed of laccase, LiP, and MnP.	[109]

ND: no data; d: days; h: hours; mo: months; 2,4-D: 2,4-dichlorophenoxyacetic acid; 2,4,5-T: 2,4,5-trichlorophenoxyacetic acid; 2,4-DCP: 2,4-dichlorophenol; PAA: phenoxyalkanoic acid; MFC: microbial fuel cell; MCPA: 2-methyl-4-chlorophenoxyacetic acid; WWTP: wastewater treatment plant; LiP: lignin peroxidase; MnP: manganese peroxidase; CYPs: cytochromes P450.

## Data Availability

No new data were created or analyzed in this study.
